# Revealing the Intrinsic Oxygen Evolution Reaction Activity of Perovskite Oxides across Conductivity Ranges Using Thin Film Model Systems

**DOI:** 10.1021/acsami.4c20141

**Published:** 2025-03-31

**Authors:** Lisa Heymann, Iris C. G. van den Bosch, Daan H. Wielens, Ole Kurbjeweit, Emma van der Minne, Ellen M. Kiens, Anton Kaus, Daniel Schön, Stephan Menzel, Bernard Boukamp, Felix Gunkel, Christoph Baeumer

**Affiliations:** † Peter Gruenberg Institute 7, Forschungszentrum Juelich GmbH, 52428 Juelich, Germany; ‡ MESA+ Institute for Nanotechnology, Faculty of Science and Technology, University of Twente, 7500 AE Enschede, Netherlands

**Keywords:** electrocatalysis, oxygen evolution reaction, conductivity, resistivity, perovskite oxide, interface layer, intrinsic catalytic activity

## Abstract

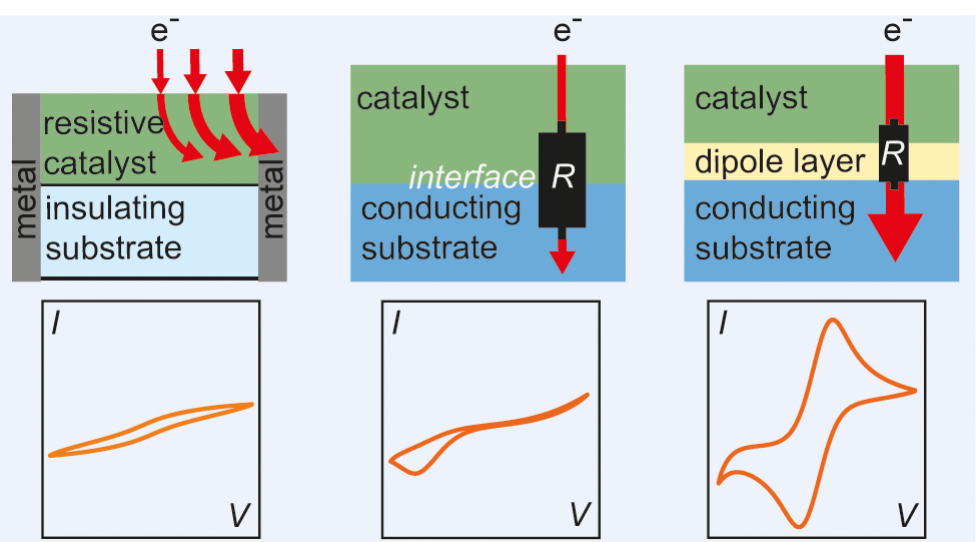

The development of efficient electrocatalysts in water electrolysis is essential to decrease the high overpotentials, especially at the anode where the oxygen evolution reaction (OER) takes place. However, establishing catalyst design rules to find the optimal electrocatalysts is a substantial challenge. Complex oxides, which are often considered as suitable OER catalysts, can exhibit vastly different conductivity values, making it challenging to separate intrinsic catalytic activities from internal transport limitations. Here, we systematically decouple the limitations arising from electrical bulk resistivity, contact resistances to the catalyst support, and intrinsic OER catalytic properties using a systematic epitaxial thin film model catalyst approach. We investigate the influence of the resistivity of the three perovskite oxides LaNiO_3-δ_ (3.7 × 10^–4^ Ω cm), La_0.67_Sr_0.33_MnO_3-δ_ (2.7 × 10^–3^ Ω cm), and La_0.6_Ca_0.4_FeO_3-δ_ (0.57 Ω·cm) on the observed catalytic activity. We tuned the electron pathway through the catalyst bulk by comparing insulating and conductive substrates. The conducting substrate reduces the electron pathway through the catalyst bulk from the millimeter to nanometer length scale. As we show, for the large electron pathways, the observed catalytic activity scales with resistivity because of a highly inhomogeneous lateral current density distribution. At the same time, even on the conducting substrate (Nb-doped SrTiO_3_), large contact resistances occur that limit the determination of intrinsic catalytic properties. By inserting interfacial dipole layers (in this case, LaAlO_3_) we lifted these interface resistances, allowing us to reveal the intrinsic catalytic properties of all examined catalysts. We find that La_0.6_Ca_0.4_FeO_3-δ_ and LaNiO_3-δ_ exhibit a similar intrinsic overpotential of 0.36 V at 0.1 mA/cm^2^, while their resistivities differ by 3 orders of magnitude. This finding shows that optimizing the electron pathway of the OER catalyst can lead the way to find new structure–activity relationships and to identify high-activity catalysts even if the electronic resistance is high.

## Introduction

Electrochemical energy conversion technologies such as electrolyzers or fuel cells are crucial for renewable energy storage, e-fuel synthesis, and a non-fossil-based industry.^1,2^ One prominent example is a water electrolyzer, where hydrogen is produced on the cathode via the hydrogen evolution reaction and oxygen is produced on the anode via the oxygen evolution reaction (OER). The optimization of catalytic processes taking place at the interface between catalyst and the electrolyte remain a challenge, particularly for the OER as it suffers from sluggish kinetics that limit the efficiency of the overall reaction.^3,4^ Therefore, the catalytic properties of the OER catalyst must be optimized to decrease the OER overpotential.^3,4^

Perovskite oxides are one promising material class to reduce the high overpotentials in the OER. They exhibit an ABO_3_ structure where the A-site is typically occupied by rare earth elements and alkaline earth elements and the B-site is occupied with transition metals.^5^ However, the electrical conductivity of perovskites varies by orders of magnitude, depending on the B-site transition metal doping level and/or the defect structure of the material.^6−8^ In the literature, it is often addressed that a high electrical conductivity, ensuring an unhindered electron pathway through the material bulk, is a crucial necessity for a good electrocatalyst.^9^ Therefore, perovskites with high resistivity are often described to be poor electrocatalysts such as LaFeO_3_, LaCoO_3_, or LaMnO_3_^10,11^ and low-conductivity perovskites have been discarded from catalyst research in some cases due to the difficulty to run sufficiently high current.^12^

However, it is hard to disentangle whether low electrochemical activity stems from high bulk resistivity or poor intrinsic catalytic properties.^13,14^ The intrinsic catalytic properties reflect the ability to lower the kinetic barriers for the electrochemical reaction at the electrolyte/catalyst interface independent of their resistivity and additional stack resistances. For example, substituting Ni into the solid-solution series of La_0.7_Sr_0.3_Fe_1–*x*_Ni_*x*_O_3-δ_ induces a phase transition and increased oxygen vacancy content leading to an overall lower resistivity that correlates with the OER performance.^15^ Furthermore, LaCoO_3_ shows comparably low OER activity in experiment, but lowering its resistivity through compressive lattice strain and introducing conductive support layers increases the OER activity.^16,17^ Additionally, contact resistances (induced, e.g., through space charge layers) at the interface to the substrate or support layer can dilute the determination of intrinsic catalytic properties.^18−22^ These examples indicate a correlation between electrical and electrochemical properties, yet it remains often unclear if a varied electrical resistivity directly affects the intrinsic catalytic activity of the OER catalyst. Further, the relations between electron transport pathways, bulk, and interface resistances remain unclear, and pathway-dependent current density losses are not quantified.

In this paper, we systematically decouple the intrinsic catalytic properties from bulk resistivity for perovskite oxides, covering a resistivity range of 3 orders of magnitude. We employ an epitaxial thin film model system approach to tune the electron transport pathway through the catalyst bulk and thin film stack. Single crystalline epitaxial thin films of perovskite oxides principally give the opportunity to reveal intrinsic properties.^23−25^ These model catalysts are free of catalyst binder and conductive carbon and exhibit smooth surface morphologies with single crystal facet orientation, allowing to determine catalytic properties also free of grain boundary effects.^23,25,26^

Here, we choose the three perovskite oxides LaNiO_3-δ_, La_0.67_Sr_0.33_MnO_3-δ_, and La_0.6_Ca_0.4_FeO_3-δ_ as epitaxial thin films in (001) orientation, exhibiting the resistivities of 3.7 × 10^–4^Ω·cm, 2.7 × 10^–3^ Ω·cm, and 0.57 Ω·cm, respectively. Metallic LaNiO_3-δ_ and La_0.67_Sr_0.33_MnO_3-δ_ are well studied electrocatalysts, especially for the OER and ORR (oxygen reduction reaction),^5,27,28^ while La_0.6_Ca_0.4_FeO_3-δ_ was previously considered as an insufficient OER catalyst.^29^ When the thin films are deposited on insulating substrates, the electron transport pathway through the bulk is several millimeters long and the observed OER activity follows the trend of the resistivities of the three perovskite oxides. To quantify inhomogeneous current density distributions, we conduct a COMSOL study, where the highly resistive La_0.6_Ca_0.4_FeO_3-δ_ shows tremendous current density variations along the catalyst/electrolyte interface. Switching to conducting Nb-doped SrTiO_3_ substrates decreases the electron transport pathway to nanometer length scales. However, large contact resistances occur between the substrate and thin films as also reported elsewhere.^22,30^ Decreasing the contact resistance via interface engineering finally allows us to reveal the intrinsic catalytic properties of the three perovskites. Surprisingly, the initially inactive and highly resistive La_0.6_Ca_0.4_FeO_3-δ_ thin film shows similarly high intrinsic OER activity as the metallic LaNiO_3-δ_, and La_0.67_Sr_0.33_MnO_3-δ_ shows the lowest intrinsic OER activity. The results show that the intrinsic catalytic activity of electrocatalysts across large conductivity ranges can be determined when an appropriate and individualized sample design is applied.

## Results

**Figure 1:**
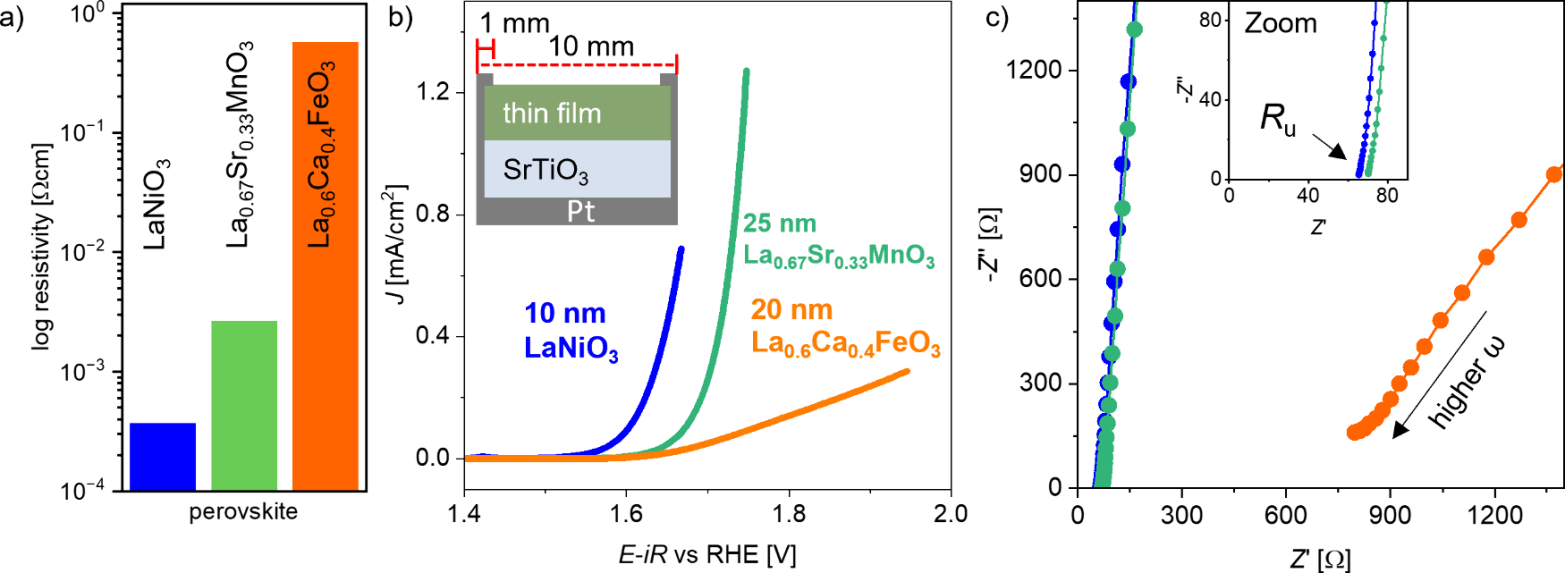
(a) Resistivity of LaNiO_3-δ_, La_0.67_Sr_0.33_MnO_3-δ_, and La_0.6_Ca_0.4_FeO_3-δ_. (b) Cyclic voltammetry of LaNiO_3-δ_, La_0.67_Sr_0.33_MnO_3-δ_ and La_0.6_Ca_0.4_FeO_3-δ_ on SrTiO_3_, with Pt side contacts to the back. The film thickness of each material is given in the legend. The CV data are *iR* corrected and averaged between anodic and cathodic sweep from the second CV cycle (see also Figure S3). The sweep rate was 10 mV/s. (c) Nyquist plots in the high frequency (ω) range of LaNiO_3-δ_, La_0.67_Sr_0.33_MnO_3-δ_, and La_0.6_Ca_0.4_FeO_3-δ_ measured at open circuit voltage (graph colors correspond to the legend in (b)). The inset shows a zoomed-in view of the Nyquist plot to the high frequency intercept of LaNiO_3-δ_ and La_0.67_Sr_0.33_MnO_3-δ_.

Epitaxial thin films of 10–25 nm thickness of LaNiO_3-δ_, La_0.67_Sr_0.33_MnO_3-δ_, and La_0.6_Ca_0.4_FeO_3-δ_ were grown by pulsed laser deposition (PLD) on single crystal (100) SrTiO_3_ substrates. The growth was tracked by reflection high energy electron diffraction (RHEED), enabling us to control the desired thin film thickness on the single unit cell level. Figure S1 shows representative RHEED data, X-ray diffraction (XRD), and atomic force microscopy (AFM), confirming the well-defined synthesis of the OER model catalysts. The surface area determined by the AFM deviates by only 1.2% between samples, which has an insignificant impact on the catalytic activity. To determine the electrical resistivity (ρ), the sheet resistance was measured in van der Pauw geometry. The resistivity of LaNiO_3-δ_ is 3.7 × 10^–4^ Ω·cm (Figure 1a) followed by La_0.67_Sr_0.33_MnO_3-δ_ with 1 order of magnitude higher resistivity (2.7 × 10^–3^ Ω·cm) and finally La_0.6_Ca_0.4_FeO_3-δ_ with 3 orders of magnitude higher resistivity (0.57 Ω·cm). The resistivity values of LaNiO_3-δ_ and La_0.67_Sr_0.33_MnO_3-δ_ grown on SrTiO_3_ are consistent with the literature.^31−33^ For a sintered La_0.6_Ca_0.4_FeO_3-δ_ ceramic, a higher resistivity of 29 Ω cm was reported.^34^ The difference between the epitaxial thin film and sintered ceramic resistivity might stem from grain boundary effects or crystal facet dependent conductivity.

Electrochemical measurements were conducted in a three-electrode configuration with a rotating disc electrode (RDE). The thin film samples were mounted on the RDE shaft as illustrated in Figure S2a, using the same approach as reported previously.^27^ For thin films deposited on insulating SrTiO_3_ substrates, Pt was sputtered on the sample back side, edges, and front side edges (see sketch in Figure S2b and Figure 1b) to provide current collection from the sides of the thin film electrocatalysts.

The OER activity of LaNiO_3-δ_, La_0.67_Sr_0.33_MnO_3-δ_, and La_0.6_Ca_0.4_FeO_3-δ_ was assessed using cyclic voltammetry (CV, Figure 1b). The CV scans were *iR* corrected with the uncompensated resistance (*R*_u_) obtained from open circuit potential (OCP) impedance spectroscopy (Figure 1c) with a linear extrapolation of the high frequency region to the *x*-axis. Note that the *iR* correction was applied to the CV curve after the electrochemical testing, as recommended^25^ for a 100% *iR* correction. The displayed CV curves are the average of the forward and backward sweeps, as illustrated in Figure S3. The current density was determined for the geometric surface area, which is the area of the inner O-ring diameter. The LaNiO_3-δ_ thin film shows the highest OER activity with an overpotential (η = *E*_OER_*– 1.23* V) of 0.37 V at 0.1 mA/cm^2^ followed by La_0.67_Sr_0.33_MnO_3-δ_ with η = 0.44 V. The La_0.6_Ca_0.4_FeO_3-δ_ thin film shows the lowest OER activity with an overpotential of 0.53 V at 0.1 mA/cm^2^. The observed OER activity is hence scaling with the resistivity of LaNiO_3-δ_, La_0.67_Sr_0.33_MnO_3-δ_ and La_0.6_Ca_0.4_FeO_3-δ_.

The CV scan of the La_0.6_Ca_0.4_FeO_3-δ_ sample exhibits a close-to-linear slope at higher current densities, suggesting that an ohmic resistance suppresses the typically expected exponential behavior. This hints at a current limitation caused by the high bulk resistivity of La_0.6_Ca_0.4_FeO_3-δ_, rather than at a limitation caused by its intrinsic catalytic activity. The presence of an additional ohmic resistance in addition to the electrolyte resistance is also apparent in the electrochemical impedance data (Figure 1c). The *R*_u_ values of LaNiO_3δ_ and La_0.67_Sr_0.33_MnO_3-δ_ are 65 Ω and 70 Ω (cf. inset in Figure 1c), while the *R*_u_ of La_0.6_Ca_0.4_FeO_3-δ_ is about an order of magnitude higher (724 Ω). Further, the imaginary part of the impedance, −*Z*″, shows a larger offset from the abscissa. This occurs because the catalyst impedance signal overlaps with the impedance signal caused by the reference electrode (see Figure S4 for a detailed discussion).^22,35^ Although the OER CV scan of La_0.6_Ca_0.4_FeO_3-δ_ (Figure 1b) was *iR* corrected with the *R*_u_ of 724 Ω, this *iR* correction appears to be insufficient to eliminate all ohmic resistances. A possible reason might be that the employed fitting procedure to determine *R*_u_ underestimates the true serial resistances in the cell, e.g., due to the large offset from the abscissa. Alternatively, the *x*-axis offset as measured in impedance spectroscopy might not include all bulk related resistances, a point to which we will return below. Note that typical *R*_u_ values are between 40 Ω and 60 Ω in 0.1 M KOH with Pt electrodes in this cell geometry (see Figure S4a), indicating that our measured *R*_u_ values contain a small contribution from sample-specific resistance (for LaNiO_3-δ_ and La_0.67_Sr_0.33_MnO_3-δ_) or are dominated by sample resistance, as is the case for La_0.6_Ca_0.4_FeO_3-δ_.

The observed scaling with resistivity may be rationalized by the sample geometry. The chosen side-contacting implies that electrons released in redox reactions at the center of the 10 × 10 mm^2^ sample must travel up to 4 mm through the bulk of the catalyst layer to reach the metal contact (Figure S2b). The comparably high resistivity of La_0.6_Ca_0.4_FeO_3-δ_ suppresses the electron transport from the reaction site at the solid–liquid interface to the metallic contacts to a larger extent than is the case for LaNiO_3-δ_ and La_0.67_Sr_0.33_MnO_3-δ_. Hence, the thin film resistivity might play a significant role in the measured OER activities.

To investigate this possible electron transport limitation in the thin film toward the metallic side contact independent of the sluggish OER kinetics, we employed CV in hexacyanoferrate K_4_[Fe(CN)_6_]/K_3_[Fe(CN)_6_] containing electrolyte, constituting the outer-sphere reversible redox couple hexacyanoferrate(II)/(III) (in the following denoted as hexacyanoferrate(II)/(III)). To obtain the typical duck shape of the CV scan, the rotation is turned off for this experiment. In contrast to inner-sphere electrocatalytic reactions like the OER, such outer-sphere fast redox couples have no significant material-dependent kinetic charge-transfer challenges but can directly reveal electronic transport limitations. For barrier-free band alignment at all involved interfaces and a low overall resistance, one expects a reversible redox process with symmetric anodic and cathodic peaks and a small peak potential separation. Any decrease in peak height as well as asymmetries can be related toward electronic resistances in the electrode stack^24^ (or in some cases toward smaller surface areas^36^). The redox potential of hexacyanoferrate(II) to hexacyanoferrate(III) is 1.2 V vs RHE, which is similar to the theoretical OER redox potential, giving the advantage to test electron transport limitations in the relevant potential range.^24^

**Figure 2:**
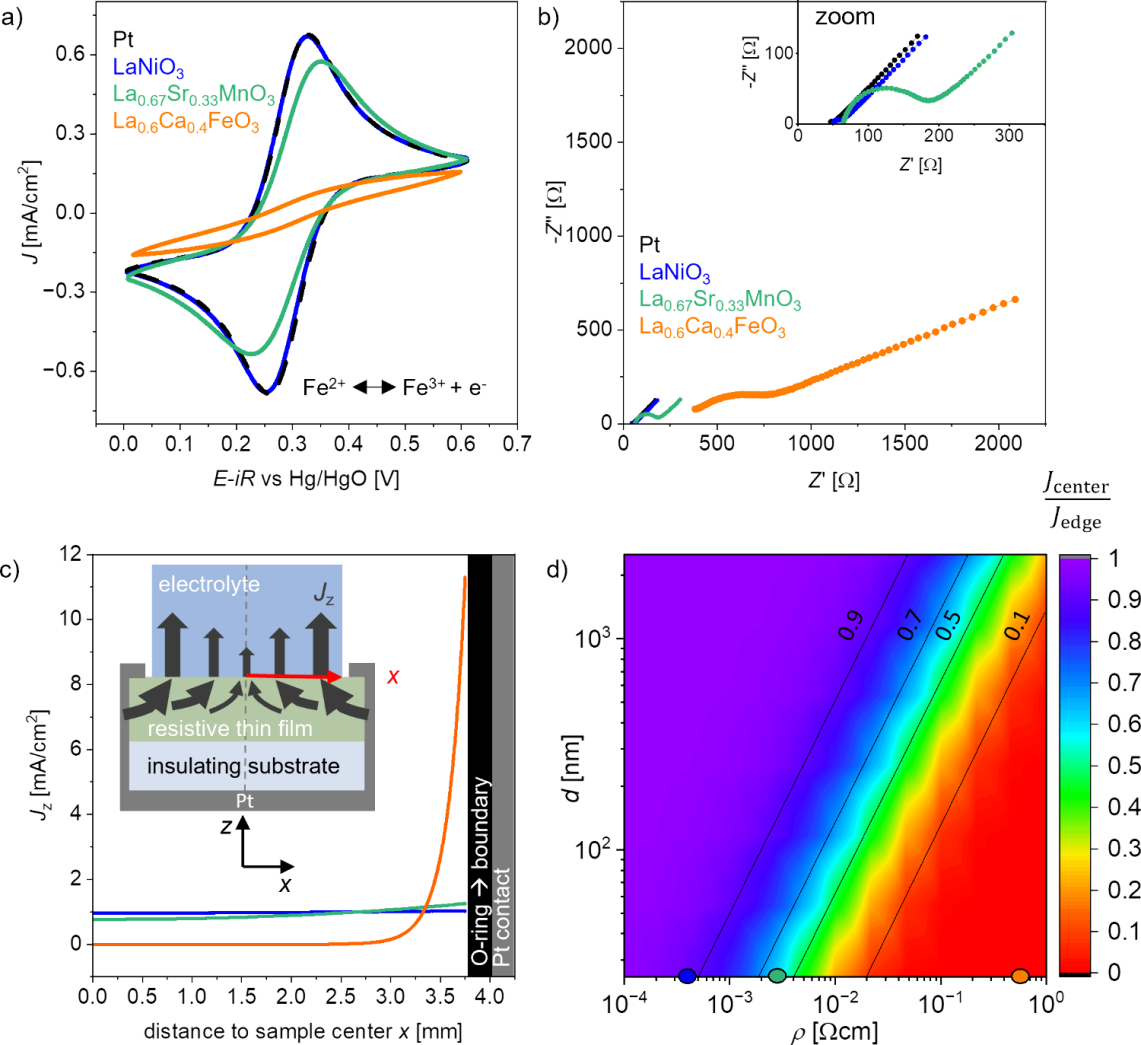
(a) CV scans of LaNiO_3-δ_, La_0.67_Sr_0.33_MnO_3-δ_, and La_0.6_Ca_0.4_FeO_3-δ_ in K_4_[Fe(CN)_6_]/K_3_[Fe(CN)_6_] solution. The sweep rate was 30 mV/s.(b) Corresponding impedance spectroscopy measured at the OCP. The inset shows a zoom to the region of 350 Ω in the *Z*′ direction. (c) Simulated current density distribution of the thin films on insulating substrates. The inset shows a sketch of the radial current density distribution *J*_*z*_(*x*) on resistive thin films along the *x*-axis of the simulation. The dashed line in the center represents the axisymmetric line cut. (d) Heat map of the current density variation  as a function of catalyst layer resistivity ρ and thickness *d*. Blue, green, and orange dots are the data points for 25 nm thick LaNiO_3-δ_, La_0.67_Sr_0.33_MnO_3-δ_, and La_0.6_Ca_0.4_FeO_3-δ_ thin films, respectively.

We compared the hexacyanoferrate(II)/(III) CV scans of 25 nm thick LaNiO_3-δ_, La_0.67_Sr_0.33_MnO_3-δ_, and La_0.6_Ca_0.4_FeO_3-δ_ thin films to a Pt thin film acting as an ideally metallic reference (Figure 2a). LaNiO_3-δ_ and Pt exhibit strongly overlapping CV curves, indicating that the electron transport to the side contacts of the LaNiO_3-δ_ thin film is not limiting and the redox reaction exhibits no significant overpotential for LaNiO_3-δ_. La_0.67_Sr_0.33_MnO_3-δ_ shows a slightly increased overpotential for the oxidation and reduction of hexacyanoferrate(II)/(III), as indicated by the slight decrease and shift of the current density maximum toward higher and lower potentials, respectively. In contrast, La_0.6_Ca_0.4_FeO_3-δ_ exhibits no clear oxidation and reduction peaks in this potential range, indicating that its high resistivity strongly limits the electron transport.

As can be seen in Figure 2b, the corresponding impedance spectra recorded at the OCP in hexacyanoferrate(II)/(III) containing electrolyte show a similar behavior. LaNiO_3-δ_ and Pt exhibit comparable spectra, whereas La_0.67_Sr_0.33_MnO_3-δ_ exhibits a larger semicircle in the high frequency range. In the low frequency range, the impedance shows a nearly linear increase at an angle close to 45° for the Pt, LaNiO_3-δ_, and La_0.67_Sr_0.33_MnO_3-δ_ thin films, indicating the presence of a semi-infinite Warburg element^37^ in the equivalent circuit. This feature stems from the mass transport limitations of the hexacyanoferrate(II)/(III) species at the solid/liquid interface. The slight overpotentials observed for La_0.67_Sr_0.33_MnO_3-δ_ in the CV scan in Figure 2a could stem from a higher resistivity compared to LaNiO_3-δ_, but the observed semicircle in the impedance indicates that there could be a significant interface barrier for the La_0.67_Sr_0.33_MnO_3-δ_ thin film reducing the observed currents as well. For La_0.6_Ca_0.4_FeO_3-δ_, *R*_u_ exhibits a significantly higher value since the higher total resistance of the thin film might add up to the overall observed *x*-axis offset, similar to the behavior of the impedance shown in Figure 1c. Moreover, the observed semicircle in the high frequency range is deformed, a characteristic often represented by a constant phase element in equivalent electrical circuits. The low-frequency region shows a distorted incline with a slope of less than 45°, which indicates that the condition for an ideal Warburg element is not fulfilled either. Together, CV and impedance spectroscopy with the hexacyanoferrate(II)/(III) redox couple confirm that thin films with a high resistivity exhibit poor electrochemical performance due to impeded electron transport in the film.

To investigate and quantify the influence of the thin film resistivity on the local current density along the thin film/electrolyte interface, a COMSOL Multiphysics study was conducted for the LaNiO_3-δ_, La_0.67_Sr_0.33_MnO_3-δ_, and La_0.6_Ca_0.4_FeO_3-δ_ films, which is illustrated in Figure 2c. In the COMSOL simulation, a fixed current of 0.44 mA was applied, corresponding to an average current density of 1 mA/cm^2^ as the exposed geometric surface area is 0.44 cm^2^. We simulated the current density distribution *J*_*z*_(*x*) across the catalyst surface from the sample center to the boundary of the catalyst, i.e., the location of the O-ring at 3.75 mm distance from the sample center. The metallic contacts are located in 4 mm distance to the sample center, approximating the real sample geometry in the RDE setup. *J*_*z*_(*x*) is the current density perpendicular to the catalyst surface, with read-out along the sample interface in the *x*-direction (further details of the simulation can be found in the Methods section and in Figure S5). Our simulation focuses on the current distribution resulting from varying catalyst conductivity, assuming sufficient electrolyte conductivity to enable homogeneous current distribution for metallic electrodes. This is justified for the treatment of the OER, which is always characterized by slow electrode kinetics. These slow kinetics are imposed in the simulation via an additional resistance at the electrode interface,^38^ representing the OER charge transfer resistance.

The resulting current density distribution shows significant differences among the three materials with varying resistivities. For LaNiO_3-δ_, the current density is almost homogeneously distributed, indicating that the low resistivity does not significantly affect the current pathway through the thin film toward the metallic contacts, and the entire catalyst area contributes similarly to the reaction current. In the sample center, the current density is 0.96 mA/cm^2^ and increases to 1.04 mA/cm^2^ at the catalyst boundary. To describe the current density variation along the sample profile, we define the ratio , which is the current density at the sample center in relation to the catalyst boundary:
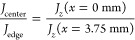


For LaNiO_3-δ_,  is about 0.92. For La_0.67_Sr_0.33_MnO_3-δ_, however, the current density in the sample center is only 0.76 mA/cm^2^, which is ∼20% lower than the value obtained for LaNiO_3-δ_. The current density increases continuously from the sample center toward the catalyst boundary reaching a current density of around 1.25 mA/cm^2^. Hence, the 1 order of magnitude higher resistivity in the La_0.67_Sr_0.33_MnO_3-δ_ film leads to a significant change of the current density distribution along the interface, where  is only 0.6. This behavior is even more pronounced for La_0.6_Ca_0.4_FeO_3-δ_. Here, the current density is only 3.6 nA/cm^2^ in the sample center and essentially does not contribute to the overall reaction. At a 3 mm radial distance, the current density increases to 0.15 mA/cm^2^. And only toward the edge, the current density increases drastically to more than 11 mA/cm^2^ leading to an extremely small  ratio of only 2 × 10^–8^. Due to the high resistance of the La_0.6_Ca_0.4_FeO_3-δ_ thin film, the area close to the metal contacts hence overproportionally contributes to the current and shows a locally higher current density (as sketched in the inset of Figure 2c). In the Supplementary Note N1, the possible inhomogeneities caused by electrolyte effects^38^ are estimated for our setup, where the variation is only about 10% in the sample center. However, according to our COMSOL simulations, the inhomogeneities caused by the thin film catalyst resistivity can reach values that are orders of magnitude higher. Hence, inhomogeneities caused by electrolyte effects have a minor influence.

Hence, the COMSOL study shows that the effective surface area accessible for electrochemical reactions is extremely small for La_0.6_Ca_0.4_FeO_3-δ_ in the chosen sample geometry. Therefore, for samples with higher resistivity, the observed electrochemical currents can effectively not be related to their geometric surface area that is exposed to the electrolyte, making it impossible to reveal their intrinsic catalytic properties in any electrochemical reaction in such sample geometry. The observed low activity of La_0.6_Ca_0.4_FeO_3-δ_ (Figure 1b) thus results from ill-defined normalization of the current density with respect to the total exposed catalyst area.

To determine which resistivity–thickness relations are suitable to reveal the intrinsic properties for this sample geometry, we extended the COMSOL study for the full resistivity range from 10^–4^ to 1 Ω·cm and thin film thicknesses of up to 2.5 μm. The expected current density ratio  is determined for various combinations of catalyst resistivity and layer thickness, yielding the heat map shown in Figure 2d. The values for 25 nm thick LaNiO_3-δ_, La_0.67_Sr_0.33_MnO_3-δ_, and La_0.6_Ca_0.4_FeO_3-δ_ thin films are marked as blue, green, and orange dots, respectively.

We consider the LaNiO_3-δ_ case with  = 0.92 as an acceptable scenario to extract intrinsic catalytic properties in this geometry, while for La_0.67_Sr_0.33_MnO_3-δ_ with  = 0.6 the current density would already be too inhomogeneous for a proper analysis. However, with an exemplary sample thickness of more than 200 nm,  would be 0.9 or more also for La_0.67_Sr_0.33_MnO_3-δ_, enabling us to principally reveal the intrinsic catalytic properties of La_0.67_Sr_0.33_MnO_3-δ_ in this sample geometry. For La_0.6_Ca_0.4_FeO_3-δ_, however, even a film thickness of 1 μm would only lead to  = 0.2, making it impossible to determine the intrinsic catalytic properties in this sample geometry. Figure S6 shows the heat map on a logarithmic scale, highlighting the extremely small  values for resistivities above 2 × 10^–2^ Ω·cm.

We emphasize that even though the trends presented in Figure 2d will be valid for any geometrical setup, i.e.,  increases with increasing film thickness or decreasing film resistivity, the absolute values of  depend also on the lateral geometry of the experimental setup. If the setup deviates greatly from that used here, additional simulations would be required to accurately estimate the variation in the current density across the interface between the thin film and the electrolyte. Nevertheless, the heat map can be used as qualitative guidance for any thin film geometry applied to the OER catalysis. We also note that ohmic drops in the solution can lead to an additional inhomogeneity. While the expected variation in current density across the electrode surface caused by electrolyte effects is much smaller than the effects observed for our highly resistive catalysts, it would be necessary to explicitly include the electrode kinetics model developed by Newman^38^ in simulations for high-resistivity catalysts for mass-transfer limited reactions, where the electrolyte effects become more relevant.

The previously mentioned ill-defined normalization of the current density in La_0.6_Ca_0.4_FeO_3-δ_ can also explain the deformed impedance features observed in Figure 2b. As the current–potential distribution exhibits a strong radial distribution, the equivalent electric circuit needs to be described as a series of *R*_u_–*RC* elements that vary along the solid/liquid interface. Such 2D surface distributions are experimentally observed as a constant phase element in the global impedance,^39−41^ thus explaining the suppressed impedance feature in the high and low frequency ranges observed for La_0.6_Ca_0.4_FeO_3-δ_. An additional reason for the non-ideal Warburg element in Figure 2b could be sideward diffusion of ionic species in the electrolyte, which has been observed as an edge effect^42^ that can principally also occur on metallic electrodes. The phenomenon of varied radial current–potential distribution could also occur in the catalyst bulk where not solely ohmic resistances are present but also capacitive contributions^43,44^ might be present in the lateral dimension. Hence, the catalyst bulk might also be described as 2D distributed *RC* elements along the lateral dimension. Therefore, *R*_u_ determined by the *x*-axis intercept of the impedance at high frequencies does not include all relevant bulk-related impedances. In other words, each location on the sample surface exhibits a different ‘effective’ *iR*_u_ value, resulting from increasing series resistances from the edge to the center. This might explain the fact that the linear slope observed for La_0.6_Ca_0.4_FeO_3-δ_ in Figure 1a cannot be compensated by a classic *iR*_u_ correction.

**Figure 3:**
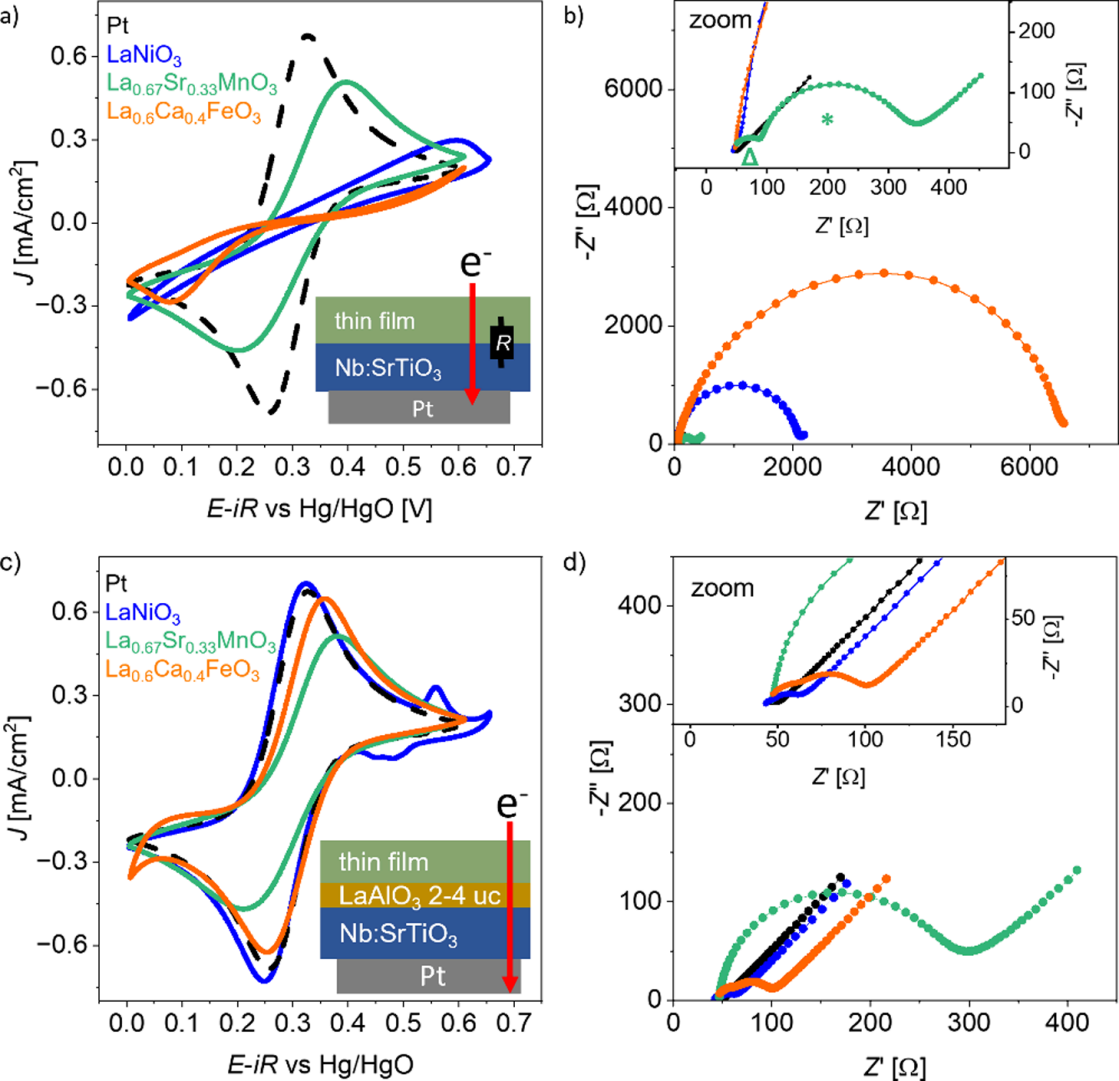
(a) CV in hexacyanoferrate(II)/(III) electrolyte for LaNiO_3-δ_, La_0.67_Sr_0.33_MnO_3-δ_, and La_0.6_Ca_0.4_FeO_3-δ_ thin films grown on Nb:SrTiO_3_ in comparison to the platinum sample. The sweep rate was 30 mV/s. (b) Corresponding impedance spectra at the OCP. Legend corresponds to (a). Inset shows the zoom of the lower impedance values. Δ refers to the semicircle in the high frequency range of La_0.67_Sr_0.33_MnO_3-δ_ and * refers to the semicircle toward lower frequencies. (c) CV in hexacyanoferrate(II)/(III) electrolyte for Nb:SrTiO_3_/2 uc LaAlO_3_/LaNiO_3δ_, Nb:SrTiO_3_/2 uc LaAlO_3_/La_0.67_Sr_0.33_MnO_3-δ_ and Nb:SrTiO_3_/4 uc LaAlO_3_/La_0.6_Ca_0.4_FeO_3-δ_ thin films in comparison to the platinum sample. The sweep rate was 30 mV/s. For LaNiO_3-δ_, additional oxidation and reduction peaks are seen at 0.55 and 0.45 V vs Hg/HgO, respectively, representing the Ni^2+^/Ni^3+^ redox reaction. (d) Corresponding impedance spectra at the OCP. Inset shows the zoom to the lower impedance values.

To suppress electron transport limitations and the large radial current density distribution of the resistive thin film electrocatalysts, the chosen OER catalyst layers were deposited on 0.5 wt % Nb-doped SrTiO_3_ (Nb:SrTiO_3_), which possesses metallic conductivity in the bulk. As sketched in Figure 3a, the electrons can now travel from the solid/liquid interface directly through the only nanometers thick films into the Nb:SrTiO_3_ substrate, which can act as a current collector. The film thickness is 10 nm for LaNiO_3-δ_, 20 nm for La_0.6_Ca_0.4_FeO_3-δ_, and 25 nm for La_0.67_Sr_0.33_MnO_3-δ_, which is far above the thickness where finite size phenomena occur. Here, Pt is sputtered only on the back side of the substrate. In this contacting geometry, La_0.6_Ca_0.4_FeO_3-δ_ and notably also LaNiO_3-δ_ show largely suppressed current densities and large peak separations in the hexacyanoferrate(II)/(III) redox reaction (Figure 3a), while the current density of La_0.67_Sr_0.33_MnO_3-δ_ is the closest to the Pt reference and the peak separation is much smaller. This indicates that although the traveling distance has changed from a few millimeters in lateral dimensions to a few nanometers in vertical dimension in this sample geometry, the charge transport remains limited. This is particularly observed for the OER catalyst with the lowest (La_0.6_Ca_0.4_FeO_3-δ_) and largest (LaNiO_3-δ_) conductivity, indicating a departure from the systematic scaling with electrical conductivity as observed before. Instead, the observed behavior suggests an interfacial contact resistance across the substrate/thin film interface,^45−48^ resulting from Schottky-type space charge layers.

For a Schottky barrier (the catalyst work function is higher than the Nb:SrTiO_3_ electron affinity), it is expected that the electron transport is more hampered for the oxidation reaction rather than for the reduction reaction.^24^ For La_0.6_Ca_0.4_FeO_3-δ_, the oxidation and reduction peaks of hexacyanoferrate(II)/(III) are asymmetric, indicating that the (Schottky-barrier-type) contact resistance at the Nb:SrTiO_3_/thin film interface especially hampers the oxidation from hexacyanoferrate(II) to hexacyanoferrate(III). A similar interface resistance is expected for LaNiO_3-δ_, which has a work function larger than the Nb:SrTiO_3_ electron affinity, as described elsewhere.^30,45,46^ However, the Ni oxidation takes place in the same measured potential window^27^ as the Fe oxidation, therefore the Ni^3+^ and Fe^3+^ formation might lead to additional asymmetric behavior in the anodic sweep compared to the cathodic sweep seen in Figure 3a.

The Nyquist plots in Figure 3b show large semicircles for La_0.6_Ca_0.4_FeO_3-δ_ and LaNiO_3-δ_ with a real impedance (*Z*′) of up to several kΩ, which may be attributed to the substrate/thin film contact resistance. In contrast, La_0.67_Sr_0.33_MnO_3-δ_ exhibits two semicircles that are below 400 Ω (marked with a green * and Δ in the inset of Figure 3b), indicating a significantly lower contact resistance at the substrate/thin film interface, consistent with the smaller peak separation and higher current densities in the hexacyanoferrate(II)/(III) redox reaction compared to La_0.6_Ca_0.4_FeO_3-δ_ and LaNiO_3-δ_. The semicircle in the high frequency range (marked with Δ) of La_0.67_Sr_0.33_MnO_3-δ_ could stem from the Nb:SrTiO_3_/thin film interface impedance, and the semicircle toward lower frequencies (*) could stem from La_0.67_Sr_0.33_MnO_3-δ_-specific and contact-independent behavior as the same order of magnitude impedance was already observed on the insulating substrate (Figure 2b).

The Nyquist plots in Figure 3b show that *R*_u_ is now in the same range of 40–50 Ω for all three materials, indicating that the additional ohmic resistance of the resistive La_0.6_Ca_0.4_FeO_3-δ_ thin film that was observed in Figure 2b is circumvented in this geometry. Nevertheless, the large contact resistance to the substrate, especially for La_0.6_Ca_0.4_FeO_3-δ_ and LaNiO_3-δ_, still affects the electrochemical performance and thus hinders the observation of intrinsic catalytic properties of the OER.

To decrease the contact resistance between the thin film and Nb:SrTiO_3_, a 2–4 uc (unit cell) thick interlayer of LaAlO_3_ was introduced (see the sketch in Figure 3c; corresponding RHEED data are shown in Figure S7). This polar oxide layer induces an electrical dipole which can counteract interfacial space charge layers and Schottky barriers, facilitating the electron transport across the interface.^21,30,49^ We note that the ideal dipole layer thickness to compensate for the built-in potentials is specific to the materials at the interface. For the LaNiO_3δ_ /Nb:SrTiO_3_ interface, 2 unit cells (uc) of LaAlO_3_ as interlayer effectively decreased the contact resistance, as evident by the resulting CV scan in the hexacyanoferrate(II)/(III) redox couple (Figure 3c) and the impedance at OCP (Figure 3d), both of which are now similar to the Pt reference (Figures 3c and 3d). This shows a clear improvement in comparison to the LaNiO_3-δ_/Nb:SrTiO_3_ sample without the LaAlO_3_ interlayer (Figure 3a and 3b).

In the case of La_0.67_Sr_0.33_MnO_3-δ_, introducing 2 uc of LaAlO_3_ results in a slightly smaller peak separation as can be seen in the hexacyanoferrate(II)/(III) CV scan (comparing Figures 3a and 3c with 0.2 and 0.17 V peak separation, respectively) but still exhibits a small remaining overpotential. Comparing the impedance data of La_0.67_Sr_0.33_MnO_3-δ_ with and without a LaAlO_3_ interlayer on Nb:SrTiO_3_ in Figures 3b and 3d shows that with the LaAlO_3_ interlayer, the semicircle in the high frequency range (Δ) is not visible anymore but the semicircle marked with a * in Figure 3b remains. The LaAlO_3_ interlayer might have compensated for the smaller interface resistance at the Nb:SrTiO_3_/La_0.67_Sr_0.33_MnO_3-δ_ interface, but an additional La_0.67_Sr_0.33_MnO_3-δ_-specific impedance behavior remains, which was observed in all contacting geometries.

For La_0.6_Ca_0.4_FeO_3-δ_, a LaAlO_3_ interlayer thickness of 4 uc was required to sufficiently decrease the Nb:SrTiO_3_/La_0.6_Ca_0.4_FeO_3-δ_ contact resistance. For the Nb:SrTiO_3_/LaAlO_3_/La_0.6_Ca_0.4_FeO_3-δ_ stack, the reduction peak from hexacyanoferrate(III) to hexacyanoferrate(II) shows now similar behavior as the Pt sample (Figure 3c) and the oxidation peak exhibits only a small overpotential. The total impedance was reduced by 2 orders of magnitude by the 4 uc thick LaAlO_3_ interlayer, comparing the impedance in Figure 3b and 3d. Only two small semicircles remain that are observed in the Nyquist plot in Figure 3d. The small remaining overpotential of the oxidation reaction might stem from a small remaining contact resistance at the Nb:SrTiO_3_/LaAlO_3_/La_0.6_Ca_0.4_FeO_3-δ_ interface and/or from an La_0.6_Ca_0.4_FeO_3-δ_-specific resistance. As a result, the electrochemical performance in this geometry should not be strongly limited by electronic transport anymore, despite the high resistivity of La_0.6_Ca_0.4_FeO_3-δ_. This is evidenced by the high currents obtained with the outer-sphere fast redox couple. This indicates that electronic transport limitations are also negligible for electrocatalytic inner-sphere redox reactions such as OER, implying that the measured electrocatalytic current in this geometry directly scales with the intrinsic ability of La_0.6_Ca_0.4_FeO_3-δ_ to catalyze the reaction of interest, here the OER.^24^

**Figure 4:**
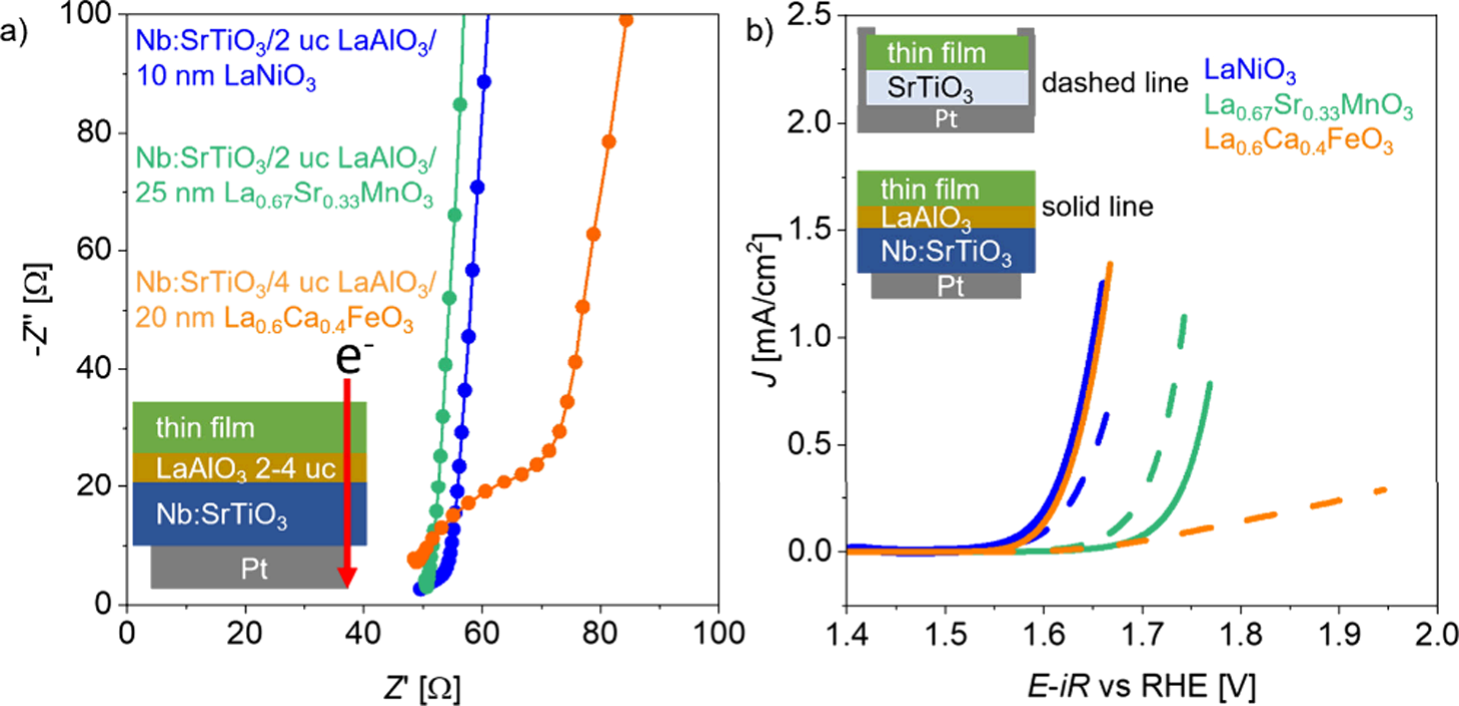
(a) Impedance spectra in the high frequency range at the OCP for the thin films on Nb:SrTiO_3_ with a LaAlO_3_ interlayer recorded in 0.1 M KOH solution. (b) OER catalytic activity of LaNiO_3-δ_, La_0.67_Sr_0.33_MnO_3-δ_, and La_0.6_Ca_0.4_FeO_3-δ_ thin films in 0.1 M KOH for the two different contacting geometries sketched in the top left corner. OER performance of the insulating SrTiO_3_ substrates is shown as dashed lines (reproduced from Figure 1c for ease of comparison), and the OER activities of Nb:SrTiO_3_/2 uc LaAlO_3_/LaNiO_3-δ_, Nb:SrTiO_3_/2 uc LaAlO_3_/La_0.67_Sr_0.33_MnO_3-δ_, and Nb:SrTiO_3_/4 uc LaAlO_3_/La_0.6_Ca_0.4_FeO_3-δ_ stacks are shown as solid lines. The sweep rate was 10 mV/s.

Hence, the three perovskites grown on Nb:SrTiO_3_ with LaAlO_3_ as the charge compensating interlayer were tested in 0.1 M KOH to reveal the intrinsic catalytic activity in the OER with minimized electron transport limitations through the bulk and substrate interface resistance. Note that in contrast to the experiments in hexacyanoferrate(II)/(III) containing electrolyte, RDE rotation is set to 1600 rpm for OER experiments to remove evolving oxygen gas from the surface during the CV scan. Figure 4a shows the corresponding impedance in 0.1 M KOH of the Nb:SrTiO_3_/LaAlO_3_/catalyst stacks. The *R*_u_ is small (40–50 Ω) for the three perovskite oxides, indicating no significant ohmic losses through bulk resistivities are present, which is consistent with the observation of the impedance in the hexacyanoferrate(II)/(III) containing electrolyte. For La_0.6_Ca_0.4_FeO_3-δ_, there is a small semicircle observed (∼20 Ω) in the high frequency range, which can stem from a small remaining contact resistance at the La_0.6_Ca_0.4_FeO_3-δ_/LaAlO_3_/Nb:SrTiO_3_ interface. The equivalent circuit and corresponding impedance fit are shown in Figure S9. Overall, however, the *R*_u_ and the contact resistances to the substrate show significantly lower absolute values than those observed in the initial in-plane geometry and LaAlO_3_ free thin film stacks on Nb:SrTiO_3_, indicating a successful removal of the OER-performance limiting current paths.

Figure 4b shows the comparison of the OER activity of the two different contacting geometries, SrTiO_3_/catalyst and Nb:SrTiO_3_/LaAlO_3_/catalyst. The SrTiO_3_/LaNiO_3-δ_ and Nb:SrTiO_3_/LaAlO_3_/LaNiO_3-δ_ stacks show a similar η of 0.37 and 0.36 V at 0.1 mA/cm^2^, respectively. La_0.67_Sr_0.33_MnO_3-δ_ shows a higher η than LaNiO_3-δ_ in both contacting geometries with η = 0.44 and 0.48 V at 0.1 mA/cm^2^ for the SrTiO_3_/La_0.67_Sr_0.33_MnO_3-δ_ and Nb:SrTiO_3_/LaAlO_3_/La_0.67_Sr_0.33_MnO_3-δ_ stacks, respectively. The deviation between the samples can occur because of small remaining contact resistances to the Nb:SrTiO_3_ substrate or because of sample-to-sample deviation. La_0.6_Ca_0.4_FeO_3-δ_ exhibits very high η on the insulating substrate (0.53 V at 0.1 mA/cm^2^, as already introduced in Figure 1b), but the Nb:SrTiO_3_/LaAlO_3_/La_0.6_Ca_0.4_FeO_3-δ_ stack shows a remarkably low η of 0.36 V at 0.1 mA/cm^2^, which is similar to that of LaNiO_3-δ_. To determine whether the CV curves shown in Figure 4b are dominated by resistances or the OER kinetics, the Tafel plots are compared in Figure S8. They show that La_0.6_Ca_0.4_FeO_3-δ_ deposited on insulating SrTiO_3_ strongly deviates from Tafel-like behavior, reaching values above 500 mV/dec, while on Nb:SrTiO_3_/LaAlO_3_ stacks, all three perovskites show reasonable Tafel slope values between 40 mV/dec and 90 mV/dec. This highlights that in a well-chosen contacting geometry, *iR* correction by the *x*-axis intercept is reasonable and that we can reveal the intrinsic catalytic activity of poorly conducting electrocatalysts.

Hence, La_0.6_Ca_0.4_FeO_3-δ_ exhibits a similar intrinsic catalytic activity in the OER as LaNiO_3-δ_, although it has 3 orders of magnitude higher resistivity. In turn, La_0.67_Sr_0.33_MnO_3-δ_, exhibiting intermediate resistivity among the three tested perovskites, shows the lowest intrinsic OER catalytic activity compared to LaNiO_3-δ_ and La_0.6_Ca_0.4_FeO_3-δ_. As a result, the intrinsic OER activity of the perovskite oxides does not scale monotonically with their electronic resistivity, and in fact, even a high resistivity perovskite can exhibit high OER performance—a result that could not have been revealed without dedicated and systematic choice of the sample substrate and interfacial layers.

## Discussion

By tuning the bulk electron transport from millimeter to nanometer scales and additionally minimizing the contact resistance in the sample stack, the intrinsic catalytic properties in the OER could be revealed for LaNiO_3-δ_, La_0.67_Sr_0.33_MnO_3-δ_, and even for the highly resistive La_0.6_Ca_0.4_FeO_3-δ_. The observed OER overpotential trend changes from η(LaNiO_3-δ_) < η(La_0.67_Sr_0.33_MnO_3-δ_) < η(La_0.6_Ca_0.4_FeO_3-δ_), as observed in resistance-dominated geometry with inhomogeneous current contribution across the catalyst interface, to η(LaNiO_3-δ_) ≈ η(La_0.6_Ca_0.4_FeO_3-δ_) < η(La_0.67_Sr_0.33_MnO_3-δ_) as the intrinsic catalytic activity trend. Hence, the intrinsic properties of perovskite catalysts do not necessarily scale with their resistivity when catalyst bulk electron transport is limited to small distances. We note that it is expected that the electronic structure, covalency, and hybridization vary among the samples. Yet, the metallic LaNiO_3-δ_ and the highly resistive La_0.6_Ca_0.4_FeO_3-δ_ still show remarkably similar intrinsic OER activity, provided that the current pathways are properly controlled.

However, even small residual interface resistances like the remaining interface resistance of around 20 Ω at the Nb:SrTiO_3_/LaAlO_3_/La_0.6_Ca_0.4_FeO_3-δ_ interface might affect the overall observed overpotential, diluting the revelation of intrinsic properties. This is especially noticeable at higher current densities. For small currents such as 0.1 mA, a 20 Ω series resistance leads to a 2 mV additional ohmic overpotential, whereas for 10 mA this already leads to a 200 mV ohmic overpotential. Additionally, as such Schottky-barrier-type resistances are voltage dependent, the interface resistance might change with applied potential, which might dilute the revelation of intrinsic properties as well.

A similar OER activity trend compared to our findings for the Nb:SrTiO_3_/LaAlO_3_/thin film stacks was obtained for LaNiO_3-δ_, La_0.75_Ca_0.25_FeO_3-δ_, La_0.5_Ca_0.5_FeO_3-δ_, and La_0.5_Ca_0.5_MnO_3-δ_ at 50 μA/cm^2^ by Suntivich et al. in powder experiments (grain size 0.2–1.0 μm) with conductive carbon.^5^ In their study, a lower activity was observed also for the manganite La_0.5_Ca_0.5_MnO_3-δ_ compared to the nickelate and calcium doped ferrates (η (LaNiO_3-δ_) ≈ η (La_0.5_Ca_0.5_FeO_3-δ_) < η (La_0.5_Ca_0.5_MnO_3-δ_)). In contrast, it is also reported in the literature that especially La_0.6_Ca_0.4_FeO_3-δ_ in the solid solution series of La_1–*x*_Ca_*x*_FeO_3-δ_ has a low catalytic activity^29^ with an overpotential (at 50 μA/cm^2^) comparable to what we observed for La_0.6_Ca_0.4_FeO_3-δ_ on the insulating substrate with a long bulk electron transport pathway. Also, the previously reported Tafel slope was 242 mV/dec for La_0.6_Ca_0.4_FeO_3-δ_,^29^ exceeding reasonable OER Tafel slope values between 30 and 120 mV/dec by far, similar to our La_0.6_Ca_0.4_FeO_3-δ_ sample on the insulating substrate. One reason for the higher overpotentials and Tafel slopes observed (compared to our Nb:SrTiO_3_/LaAlO_3_/La_0.6_Ca_0.4_FeO_3-δ_ sample and to the findings of Suntivich et al.) could be a larger grain size in the catalyst powder of the highly resistive La_0.6_Ca_0.4_FeO_3-δ_. This could extend the electron transport pathway through the powder bulk significantly, even with conductive carbon, so that larger current losses occur. Additionally, conductive carbon can, for example, change the valence state of the B-site or act as a cocatalyst,^8,50,51^ hindering to reveal intrinsic catalytic properties as well.

Our findings and the comparison to the literature indicate that the establishment of catalyst design rules must be taken with care, especially when materials across large resistivity ranges are tested and substrate-to-catalyst contact resistances occur. One design rule addressed in the literature stems from the observation that a small charge transfer energy leads to higher OER activity.^11^ However, a large charge transfer energy (which is the energy distance between the occupied O 2p and first unoccupied transition metal 3d states) typically also leads to a lower conductivity, which can lead to lower measured (but not lower intrinsic) OER activity.^11^ Therefore, it is crucial not to assume a direct correlation between low conductivity and low OER catalytic activity, which has become obvious from our revelation that metal oxides with low conductivity can exhibit a high catalytic activity. Extra steps must be taken to disentangle intrinsic catalytic activity from conductivity to accurately assess performance. As we have shown, this can be achieved by using epitaxial model systems with appropriate current collectors and favorable interface properties.

## Conclusion

We have shown that highly resistive perovskite oxides can be intrinsically as active as quasi metallic electrocatalysts in the OER. We decoupled the bulk resistivity and substrate/thin film contact resistances from intrinsic catalytic processes at the solid/liquid interface for the three perovskites LaNiO_3-δ_, La_0.67_Sr_0.33_MnO_3-δ_, and La_0.6_Ca_0.4_FeO_3-δ_ by keeping the electron transport pathway through the catalyst bulk on the nanometer scale and minimizing the contact resistance to the Nb:SrTiO_3_ substrate. Through the insertion of a LaAlO_3_ dipole layer, the contact resistance between Nb:SrTiO_3_ and catalyst was strongly decreased. Thus, we could reveal that LaNiO_3-δ_ and La_0.6_Ca_0.4_FeO_3-δ_ have similar intrinsic catalytic properties, even though La_0.6_Ca_0.4_FeO_3-δ_ has 3 orders of magnitude higher resistivity compared to LaNiO_3δ_. La_0.67_Sr_0.33_MnO_3-δ_ has intrinsically a lower OER activity compared to those of La_0.6_Ca_0.4_FeO_3-δ_ and LaNiO_3-δ_. Hence, electrical conductivity does not necessarily correlate with intrinsic catalytic properties. Therefore, it is of high importance to quantify electron pathway dependent current density losses in chosen sample geometries as well as to distinguish intrinsic properties from resistivity for the establishment of OER catalyst design rules.

## Experimental Methods

### Thin Film Fabrication

The epitaxial thin films were deposited by PLD on single crystal SrTiO_3_ and Nb:SrTiO_3_ 10 × 10 mm^2^ substrates in the (100) orientation. LaNiO_3-δ_ was grown with a fluence of 1.9 J/cm^2^ with a pulse repetition rate of 2 Hz, with a target-to-substrate distance of 50 mm and with an oxygen partial pressure of 0.04 mbar at 450 °C (650 °C respectively for the sample discussed in Figures 2a and 2b; the growth temperature can have an influence on the catalytic activity but does not drastically change the conductivity that is important for the hexacyanoferrate(II)/(III) CV experiment^27^). La_0.67_Sr_0.33_MnO_3-δ_ was grown with a fluence of 2 J/cm^2^, with a pulse repetition rate of 2 Hz and target-to-substrate distance of 50 mm at a growth temperature of 750 °C and an oxygen partial pressure of 0.266 mbar. The LaAlO_3_ interlayer between Nb:SrTiO_3_ and LaNiO_3-δ_ and La_0.67_Sr_0.33_MnO_3-δ_, respectively, was grown with a fluence of 1.4 J/cm^2^, 1 Hz pulse repetition rate at a growth temperature of 650 °C, and an oxygen partial pressure of 0.002 mbar. La_0.6_Ca_0.4_FeO_3-δ_ was deposited at 650 °C, with a laser fluence of 2.2 J/cm^2^ and an oxygen partial pressure of 0.05 mbar, and the target-to-substrate distance was 55 mm. An LaAlO_3_ interlayer was grown on Nb:SrTiO_3_ at 700 °C with 1.8 J/cm^2^ laser fluence and 1 × 10^–4^ mbar oxygen partial pressure.

### Physical Characterization

AFM scans were recorded with a Cypher SPM (Research Asylum, Germany) atomic force microscope in tapping mode. XRD was conducted with a D8 ADVANCE diffractometer (Bruker AXS GmbH, Germany) that was equipped with a Cu cathode for K_α_ radiation, and the scans were recorded in 2θ-ω geometry.

### Electrochemical Characterization

To ensure electrical contact in the electrochemical cell, the samples deposited on SrTiO_3_ were sputtered with 50 nm Pt on the back side, edges, and front edges (as shown in Figure S2). Samples grown on Nb:SrTiO_3_ were sputtered only from the back side. Electrochemical measurements of the thin films were conducted with a RDE with a rotation speed of 1600 rpm in a three-electrode configuration in 0.1 M KOH. The KOH pellets were provided from Sigma-Aldrich (purity 99.99%) and dissolved in deionized water (Milli-Q, >18.2 MΩ·cm). The counter electrode was a spiraled Pt wire. The reference electrode was a Hg/HgO electrode protected from the solution by a Teflon tube filled with 1 M KOH. The thin films were mounted on the RDE with a custom-made PEEK holder. The thin film back side and front edges were sealed with an O-ring from the electrolyte. Here, the back of the substrate is mechanically pressed against the rotary shaft of the RDE. The geometric surface area that is exposed to the electrolyte is equal to the inner area of the O-ring.

To obtain the OER activity in 0.1 M KOH, the electrolyte was purged with an O_2_ gas for 30 min before and during the measurement. Before the determination of the OER activity with CV, impedance spectroscopy was recorded at the OCP and double layer capacitance measurements were conducted in the range from 0.0 to 0.1 V vs Hg/HgO (for La_0.6_Ca_0.4_FeO_3-δ_ from 0.1 to 0.2 V) with an increasing scan rate from 10 to 500 mV/s. To record the redox behavior of LaNiO_3-δ_ thin films, the potential window from 0.2 to 0.65 V vs Hg/HgO was recorded with an increasing scan rate from 10 to 500 mV/s. To determine the OER activity, CV scans were conducted from 0.2 to 1.15 V vs Hg/HgO to reach at least 0.5 mA/cm^2^. OER currents were normalized by the geometric surface area, as appropriate for epitaxial thin films^25^ and as justified by the low measured specific surface area. While powder catalyst activity is typically determined above 10 mA/cm^2^, epitaxial thin film activity determination is appropriate at a current density of 0.1 mA/cm^2^ as they exhibit such smooth and single-crystal-like surface morphologies.^25^ Hence, the OER performance was determined in a current range not exceeding 1.2 mA/cm^2^. The Hg/HgO electrode was calibrated against the RHE (HydroFlex, USA). The averaged value of 0.887 V vs Hg/HgO was used to determine the obtained voltage on the RHE scale. The impedance was recorded in a frequency range of 100 kHz to 0.1 Hz with an amplitude of 20 mV. As the uncompensated resistance is observed in the range of 10 kHz, the spectra are shown for data points from 10 kHz and below (see for clarity Figure S4). Electrochemical measurements with the hexacyanoferrate(II)/(III) redox couple were conducted in a 0.1 M KOH solution with an equimolar concentration of 0.003 mol/L of K_4_[Fe(CN)_6_] [H_2_O] and K_3_[Fe(CN)_6_] (Sigma-Aldrich, 99.5% and 99.0%). The RDE rotation was off for the experiments in the hexacyanoferrate(II)/(III) containing electrolyte. Impedance was recorded at the OCP and CV scans were recorded between 0 and 0.6 V vs Hg/HgO. For LaNiO_3-δ_, the window was extended to 0.65 V vs Hg/HgO to obtain a possible contribution from Ni oxidation.

### COMSOL Study

Simulations for the current density distribution along the film/electrolyte interface were carried out in COMSOL Multiphysics 6.2 by using the electric current (ec) module. A detailed sketch, description of the simulation, and the corresponding parameter values can be found in Figure S5. Current is injected from the Pt back electrode of the substrate and travels to the thin film edge and subsequently to the sample center with resistivities, as listed in the main text. The electrons travel through an interfacial boundary layer to the electrolyte with a resistance that was set to 100 Ω, representing typical charge transfer resistances at low applied voltage. Finally, they travel through the electrolyte to the counter electrode (ground). As the thin film has a large aspect ratio (25 nm thickness vs 3.75 cm length, i.e., the distance from the sample center to the O-ring), initial coarse simulations were carried out to optimize the geometry and mesh as much as possible. Here, we used a 2D axisymmetric geometry to further reduce the number of mesh elements. As expected, since the substrate has a very large resistance, no current flows from the Pt electrode through the substrate itself. Hence, in the final model, the substrate was removed to reduce the amount of mesh elements. A combination of free quad and boundary layer meshes was used to create a mesh with a high quality while keeping the number of meshing elements in a reasonable range (mesh sketch can be found in the Figure S5). The current densities *J*_*z*_(*x*) shown in Figure 2c are extracted by a line cut at the boundary between the thin film and the electrolyte.

## Data Availability

Data are available from the authors upon reasonable request.

## Supporting Information Available

RHEED, AFM, and XRD of LaNiO_3-δ_, La_0.67_Sr_0.33_MnO_3-δ_, and La_0.6_Ca_0.4_FeO_3-δ_ thin films; sketch of thin film placement on the RDE and sketch of O-ring placement and sputtered Pt contacts; methodology of averaging the CV forward and backward sweep; impedance spectra at high frequencies showing reference electrode contribution; COMSOL simulation sketch, parameter values, mesh, and simulation description; heat map of Figure 2d on a logarithmic scale; RHEED of 2 unit cell LaAlO_3_ deposition and subsequent LaNiO_3-δ_ deposition; Tafel plot and its first derivative of Figure 4b; and equivalent circuit and impedance fit of the Nb:SrTiO_3_/LaAlO_3_/La_0.6_Ca_0.4_FeO_3-δ_ stack in Figure 4a (PDF)
