# Supplementary material for: Revealing the Intrinsic Oxygen Evolution Reaction Activity of Perovskite Oxides across Conductivity Ranges Using Thin Film Model Systems
Source: ACS Appl Mater Interfaces. 2025 Mar 31;17(14):21110–21. doi: 10.1021/acsami.4c20141 (PMC12005614; doi:10.1021/acsami.4c20141)
Supplement: Supplementary file 1 — am4c20141_si_001.pdf [file am4c20141_si_001.pdf]

Supporting Information

Revealing the intrinsic oxygen evolution reaction activity of perovskite oxides across conductivity ranges using thin film model systems

Lisa Heymann[†,*], Iris C. G. van den Bosch[§], Daan H. Wielens[§], Ole Kurbjeweit[†], Emma van der Minne[§], Ellen M. Kiens[§], Anton Kaus[†], Daniel Schön[†], Stephan Menzel[†], Bernard Boukamp[§], Felix Gunkel[†,*], Christoph Baeumer[†,§,*]

[†]Peter Gruenberg Institute 7, Forschungszentrum Juelich GmbH, 52428 Juelich, Germany

[§]MESA+ Institute for Nanotechnology, Faculty of Science and Technology, University of Twente, Enschede, Netherlands

*Corresponding authors

Lisa Heymann: l.heymann@fz-juelich.de

Felix Gunkel: f.gunkel@fz-juelich.de

Christoph Baeumer: c.baeumer@utwente.nl

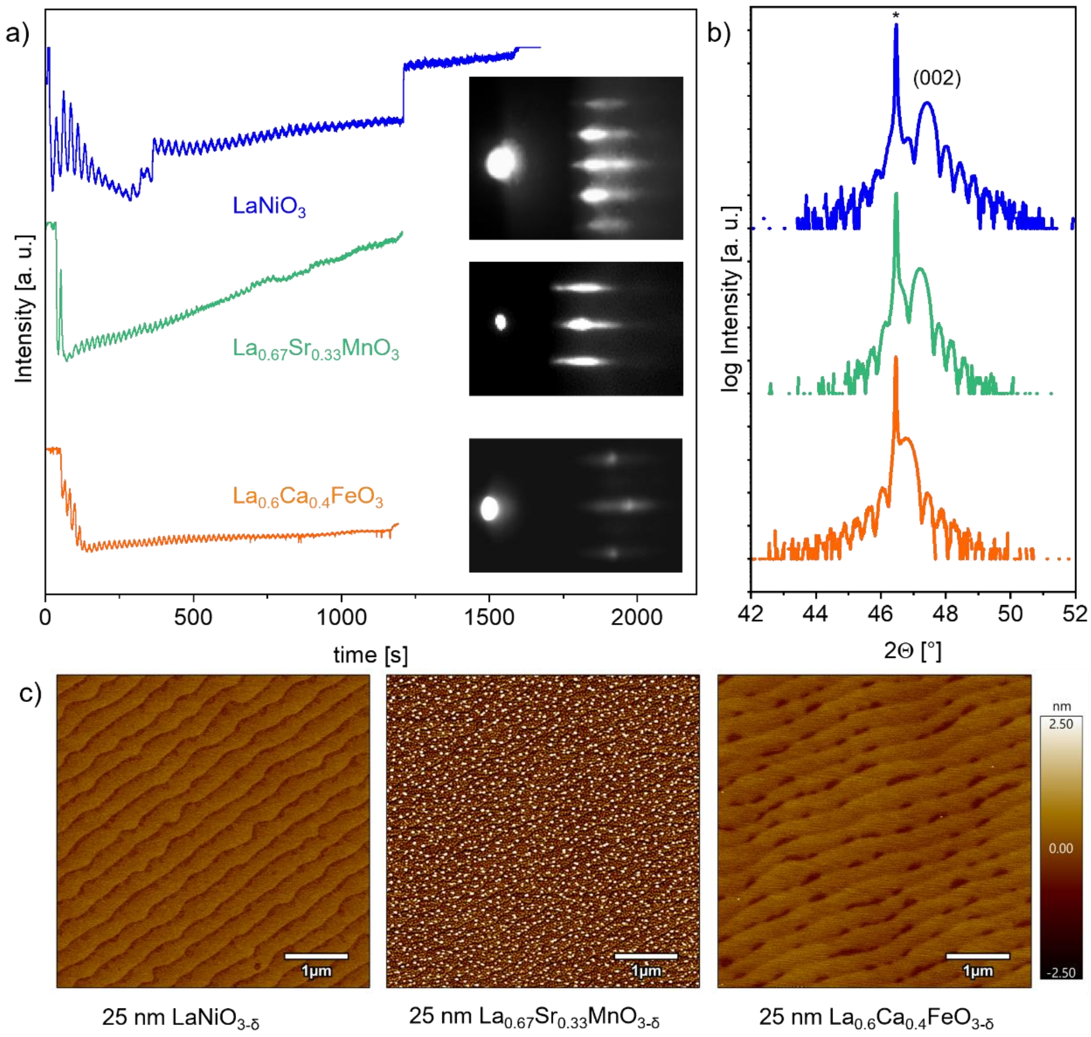

Figure S1: **a**) RHEED intensity oscillations of the specular spot for 25 nm thick $LaNiO_{3-\delta}$, $La_{0.67}Sr_{0.33}MnO_{3-\delta}$ and $La_{0.6}Ca_{0.4}FeO_{3-\delta}$ thin films deposited on $SrTiO_3$. The thin films grow in a layer-by-layer mode where in the case of $La_{0.67}Sr_{0.33}MnO_{3-\delta}$ and $La_{0.6}Ca_{0.4}FeO_{3-\delta}$, the RHEED oscillations start to fade out after 800 s. The sharp intensity increase seen for the $LaNiO_{3-\delta}$ growth stems from manually increased primary beam intensity at around 360 s and 1210 s. The corresponding RHEED patterns are shown on the right after 25 nm thin film growth. Insets: RHEED diffraction pattern. **b**) X-ray diffraction patterns of the corresponding thin films in 2θ-ω geometry. The peak marked with the * represents the diffraction signal of (002) $SrTiO_3$. The diffractograms were obtained after electrochemical treatment, confirming the high crystalline quality throughout the application. **c**) AFM scans of the corresponding thin films. $LaNiO_{3-\delta}$ and $La_{0.6}Ca_{0.4}FeO_{3-\delta}$ exhibit a smooth surface morphology with the underlying step terrace structure from the substrate. The root mean square (RMS) roughness is 270 pm for $LaNiO_{3-\delta}$ and 290 pm for $La_{0.6}Ca_{0.4}FeO_{3-\delta}$ which is comparable to a pristine $SrTiO_3$ substrate surface (~150 pm). $La_{0.67}Sr_{0.33}MnO_{3-\delta}$ exhibits particles at the surface yielding an RMS of 1.8 nm with islands of up to 7 nm height. The resulting surface areas of all films are comparable within a 2% range compared to the geometric surface area. Thus, the surface area is very similar for all three thin films which cannot significantly influence the catalytic activity, and approximating the actual surface area with the geometric surface area is justified.

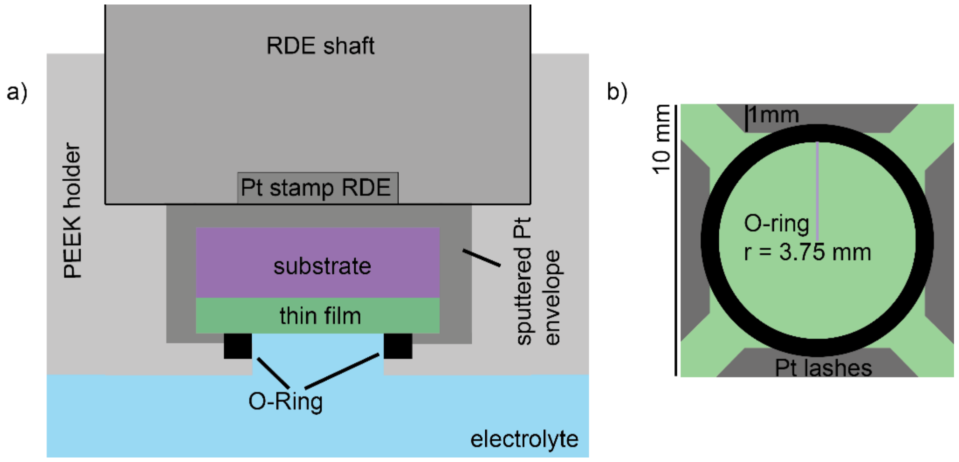

Figure S2: **a**) Sketch of a thin film mounted in the PEEK holder onto the RDE shaft. O-Ring seals the electrolyte from the sample backside and edges. The thin film is sputtered with 50 nm Pt to ensure electrical contact to the Pt stamp of the RDE. **b**) Sample geometry for electrochemical measurements. The width of sputtered Pt is 1 mm at the front edges. The sample area that is exposed to the electrolyte corresponds to the inner diameter of the O-ring (0.44 cm^2).

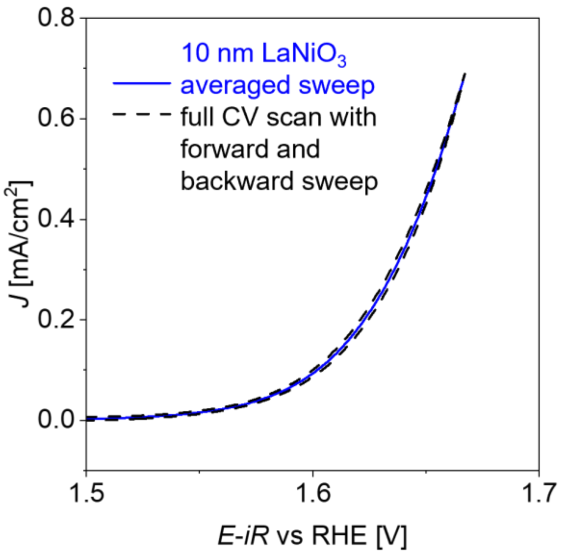

Figure S3: The OER CV scans shown in figure 1b and figure 4b are averaged from the forward and backward sweep (black, dashed lines) to correct the background from capacitive contributions[1]. The resulting averaged scan is shown in blue. Here, we show the example of $LaNiO_{3-\delta}$ on $SrTiO_3$, see main text figure 1b. The sweep rate was 10 mV/s.

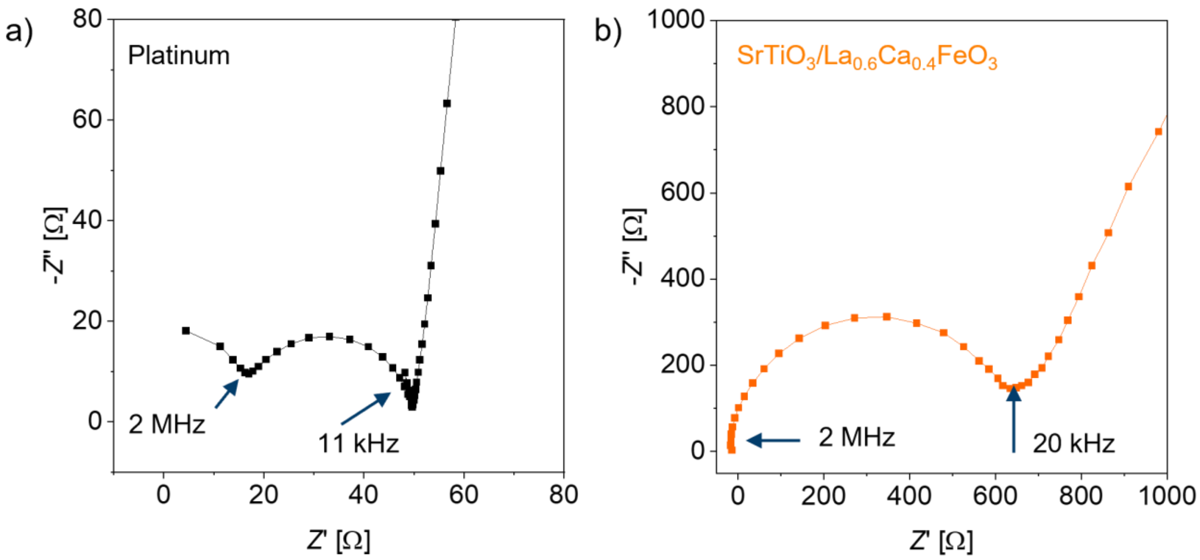

Figure S4: **a**) Nyquist plot of a Pt sample in the high frequency range. The sample was placed in 0.1 M KOH and the impedance was measured at the open circuit potential. There is a semicircle observed between 2 MHz and 11 kHz. When the reference electrode is equipped with a shunt that consists of a Pt wire and capacitor in parallel, the impedance feature systematically changes with capacitor size. Hence, the impedance feature can be attributed to the frit of the reference electrode, as was also described in the literature[2]. At 11 kHz, the imaginary part of the impedance is zero and the x-axis offset represents the uncompensated resistance R_u. At lower frequencies, a large incline is seen which is attributed to the working electrode solid/liquid interface. **b**) Nyquist plot of 25 nm $La_{0.6}Ca_{0.4}FeO_{3-\delta}$ on $SrTiO_3$ in the high frequency range. The impedance signal from the reference electrode (dominating at high frequencies) overlaps with the impedance signal of the working electrode and therefore -Z'' does not reach zero. This hinders an accurate determination of the R_u.

a)

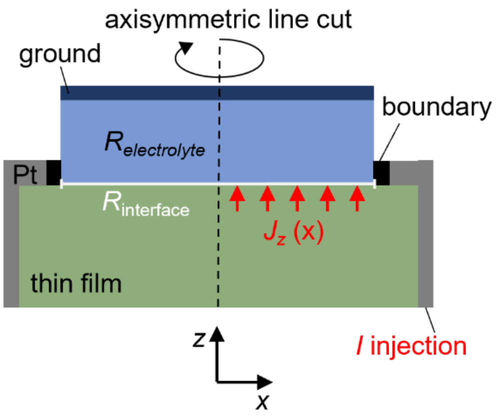

b)

Input parameters	Value/ equation
d(thin film)	25 nm
ρ(thin film)	According to fig. 1a manuscript
σ(thin film)	1/*ρ*
w(thin film)	5 mm
d(electrolyte)	10 µm
w(electrolyte)	3.75 mm
R(electrolyte)	50 Ω
σ(electrolyte)	$d_{electrolyte}/(\pi w_{\mathrm{electrolyte}}{}^{2} * R_{\mathrm{electrolyte}})$
d(interface)	5 nm
R(interface)	100 Ω
σ(interface)	$d_{\mathrm{interface}}/(\pi w_{\mathrm{electrolyte}}{}^{2} * R_{\mathrm{interface}})$
σ(Pt)	0.94x 10^7 S/m

c)

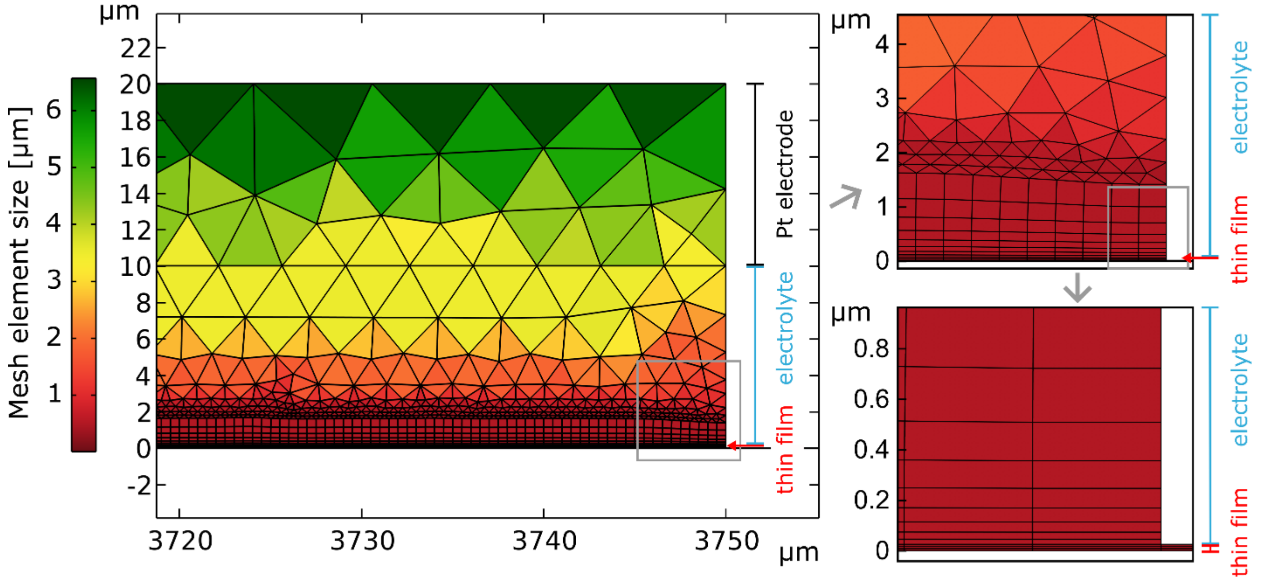

Figure S5: **a**) Sketch of the sample system geometry applied in the COMSOL study. The dashed line and circular arrow represent the axisymmetric line cut. The normal current density J_z was obtained below the sample surface in *x* direction from the sample center to the catalyst boundary (i.e. the O-ring). The electrolyte (blue box) is defined as a solid material with the resistance $R_{\mathrm{electrolyte}}$ = 50 Ω that is typically observed as uncompensated resistance (R_u) in our experiments (mainly stemming from the electrolyte resistance in 0.1 M KOH solution). The thin film/electrolyte interface resistance ($R_{\mathrm{interface}}$ = 100 Ω), i.e. the charge transfer resistance, is applied as a boundary condition directly at the interface and is marked

with the white line. Above the electrolyte layer, the ground contact (as Pt electrode) is located. **b**) Summarized simulation parameters for thicknesses (*d*), widths (*w*), resistivity (*ρ*) and conductivity (*σ*). **c**) Illustration of the 2D mesh. As the model has a very large width (5000°µm), but is very thin (20°µm), the mesh can only be visualized here using a section. Triangular and quadrilateral mesh elements are used. A comparatively large mesh can be utilized for the electrode and the electrolyte. Since the resistive film is very thin (0.025°µm), the mesh elements in the electrolyte are shrunk in the direction of the interface of the thin film. The top right graph is the zoom of the marked area in grey in the main panel. The graph in the bottom right is the zoom towards the thin film/electrolyte interface.

Simulation details

The current continuity equation $-\nabla\,\sigma\,\nabla\,\mathrm{V} = \ 0$ is solved in the electrolyte and thin film domain. σ is the conductivity and V is the potential. The whole Pt domain on top of the electrolyte is set to GND ($V = 0$), while the Pt side electrode serves as current source fulfilling

$$\oint \mathbf{nJ}\,dS = I_0,$$

where S is the surface of the domain, J is the current density and **n** the normal unit vector pointing outwards. I_0 is the applied current. All outer boundaries are considered insulating using a Neumann condition, i.e., **nJ** = 0. At the interface between electrolyte and thin film, a thin interfacial resistive layer is assumed which is modeled using the boundary condition

$$\mathbf{nJ}_{\text{electrolyte}} = \frac{\sigma_{\text{interface}}}{d_{\text{interface}} * (V_{\text{electrolyte}} - V_{\text{thin film}})}$$

and

$$\mathbf{nJ}_{\text{thin film}} = \frac{\sigma_{\text{interface}}}{d_{\text{interface}} * (V_{\text{thin film}} - V_{\text{electrolyte}})}$$

Here, $d_{\text{interface}}$ is the virtual thickness of the interfacial layer and $\sigma_{\text{interface}}$ is the interface conductivity, which is calculated according to

$$\sigma_{\text{interface}} = \frac{d}{R_{\text{interface}}\ \pi\ r_{\text{electrolyte}}^2}$$

to result in a surface resistance of $R_{\text{interface}}$ = 100 Ω. The voltages $V_{\text{thin film}}$ and $V_{\text{electrolyte}}$ are the local potentials along the interface within the thin film and the electrolyte, respectively. This condition leads to a potential jump at the thin film/electrolyte interface.

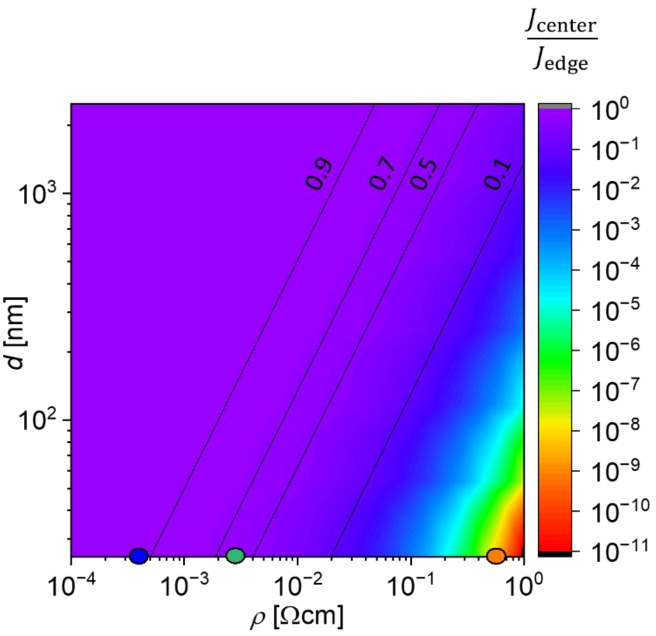

Figure S6: Heatmap of the $\frac{J_{\text{center}}}{J_{\text{edge}}}$ ratio on a logarithmic scale to obtain the rapidly increasing inhomogeneity above a resistivity of 2 10^{-2} Ωcm.

Supplementary note N1:

The purpose of the COMSOL simulations is to quantify inhomogeneities in the current distributions caused by the resistivity of the catalyst thin films. Importantly, as addressed in the manuscript, other inhomogeneities can occur from electrolyte effects where Tafel polarization dominates. According to Newman[3] the value of the term:

$$\frac{\beta ZF}{RT}\frac{r_0 i_{\text{avg}}}{\kappa_\infty}$$

estimates the extent of the inhomogeneities in this scenario. β denotes the symmetry factor of the reaction, Z is the number of charges of the redox reaction, r_0 the radius of the disk, i_{avg} the average current density and κ_∞ the conductivity of the solution. In our experiment κ_∞ is 0.02 S/cm, r_0 is 0.375 mm and i_{avg} is 1 mA/cm^2, as also taken into account in the COMSOL study. Under the assumption that $\beta = -0.5$, the term $\frac{\beta ZF}{RT}\frac{r_0 i_{\text{avg}}}{\kappa_\infty}$ yields the value 1.3. This corresponds to a rather linear inhomogeneous current density distribution along the electrode with a ratio of $\frac{i}{i_{\text{avg}}} \approx 0.9$ in the sample center whereas the inhomogeneity $\frac{i}{i_{\text{avg}}}$ caused by the thin film catalyst resistivity can be orders of magnitude higher as in the case for $La_{0.6}Ca_{0.4}FeO_{3\text{-}\delta}$ with $\frac{i}{i_{\text{avg}}} \approx 3.6 * 10^{-6}$.

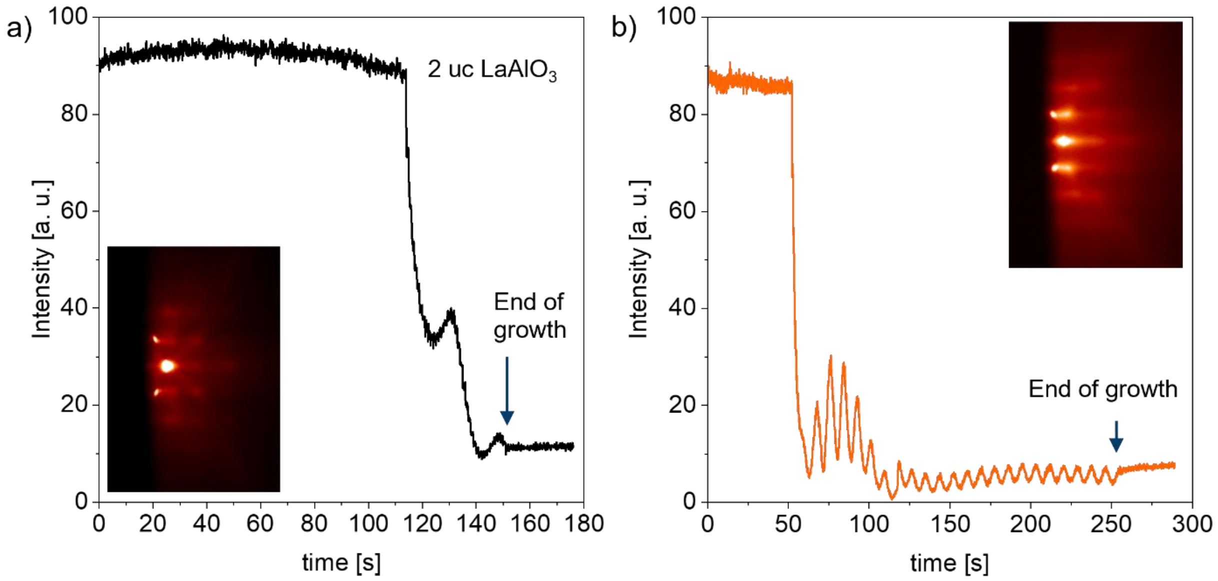

Figure S7: **a)** The growth of 2 uc $LaAlO_3$ on Nb:$SrTiO_3$. Inset: Initial RHEED pattern of the pristine substrate surface. The growth was stopped right after the second local maximum of the RHEED oscillation. **b**) Subsequent deposition of 25 uc of $LaNiO_3$ on the $LaAlO_3$ layer. Inset: Final RHEED pattern of the Nb:$SrTiO_3$/ $LaAlO_3$ 2 uc/ $LaNiO_3$ 25 uc stack, confirming high-quality growth with similar surface compared to Figure S1a).

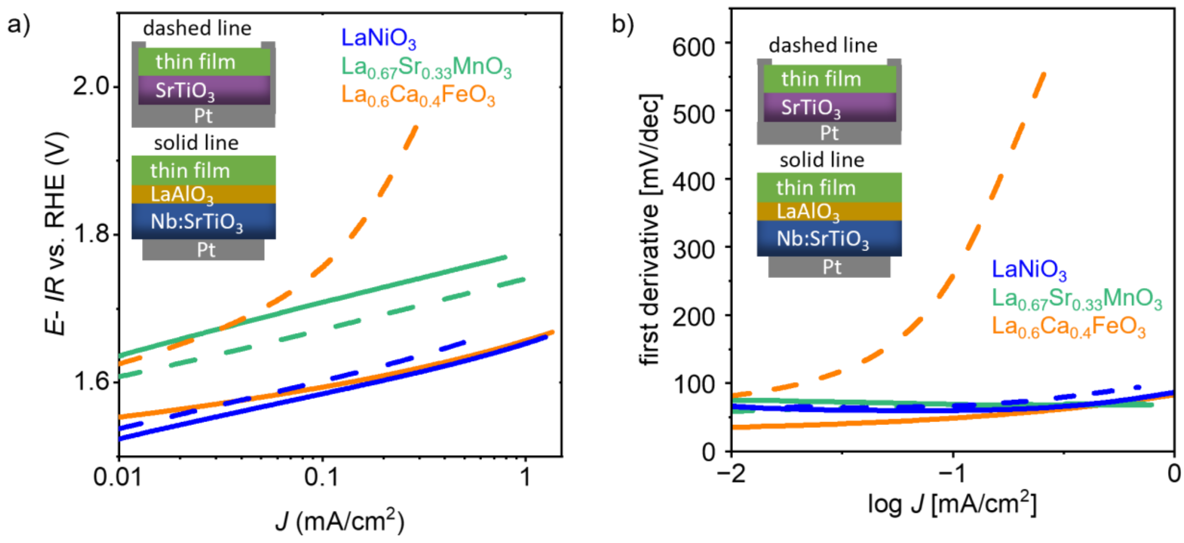

Figure S8: **a**) Tafel plot-like illustration of figure 4b in the manuscript. The thin films deposited on insulating $SrTiO_3$ with Pt side contacts are shown as dashed line, the thin films deposited on Nb:$SrTiO_3$/2-4 uc $LaAlO_3$ as solid line. **b**) Tafel slope values obtained by taking the first derivative, plotted against the logarithmic current density.

$LaNiO_{3-\delta}$ and $La_{0.67}Sr_{0.33}MnO_{3-\delta}$ show an almost linear slope in the Tafel plot in both contacting geometries. This indicates that no significant resistances hamper catalytic activity. Corresponding Tafel slope values derived by the first derivative moderately vary with the current density, yielding values between 40 mV/dec and 90 mV/dec. The Tafel slopes increase at higher current densities, as expected for similar compounds, e.g. due to changes in coverage[4,5]. This also confirms the validity of a simple *iR* correction in this current density regime for these samples.

However, the Tafel plot of $La_{0.6}Ca_{0.4}FeO_{3-\delta}$ deposited on insulating $SrTiO_3$ shows a clearly non-linear strong incline over the whole current density range, indicating that the thin film resistivity dominates the observed OER activity. The corresponding Tafel slope values seen in b) reach almost 600 mV/dec, exceeding reasonable OER Tafel slope values of 30-120 mV/dec by far. On the contrary, the $La_{0.6}Ca_{0.4}FeO_{3-\delta}$ thin film deposited on Nb:$SrTiO_3$ with a 4 uc thick $LaAlO_3$ interlayer shows very similar Tafel plot behavior as $LaNiO_{3-\delta}$ and $La_{0.67}Sr_{0.33}MnO_{3-\delta}$, highlighting that the resistance-minimized contacting geometry can reveal intrinsic catalytic properties. Here, the slight incline at higher current densities might also stem from the small interfacial resistance of ~20 Ω as explained in the discussion section of the manuscript.

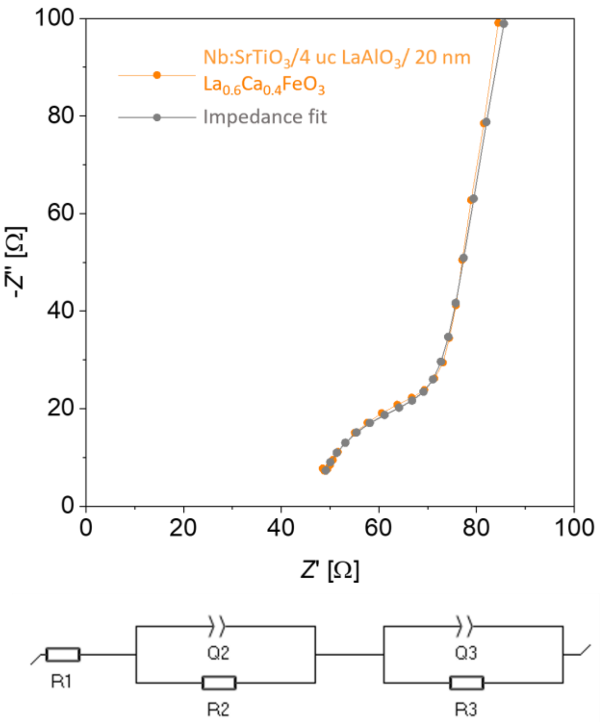

Figure S9: To estimate the remaining resistance at the Nb:$SrTiO_3$/4 uc $LaAlO_3$/20 nm $La_{0.6}Ca_{0.4}FeO_{3-\delta}$ interface shown in figure 4a of the manuscript, a *Z*-fit was conducted with the EC-Lab V 11.50 software. The equivalent electric circuit is given below the graph. R1 is the uncompensated resistance R_u. The R2/Q2 element represents the $La_{0.6}Ca_{0.4}FeO_{3-\delta}$ /electrolyte interface, which is seen in the Nyquist plot as the increasing impedance towards lower frequencies. The R3/Q3 element represents the Nb:$SrTiO_3$/4 uc $LaAlO_3$/20 nm $La_{0.6}Ca_{0.4}FeO_{3-\delta}$ interface. The *Z*-fit of the given circuit is shown in grey in the graph. According to the *Z*-fit, R1 (R_u) = 47 Ω and R3 = 25 Ω.

References

(1) Wei, C.; Rao, R. R.; Peng, J.; Huang, B.; Stephens, I. E. L.; Risch, M.; Xu, Z. J.; Shao-Horn, Y. Recommended Practices and Benchmark Activity for Hydrogen and Oxygen Electrocatalysis in Water Splitting and Fuel Cells. *Advanced Materials* **2019**, *31* (31), 1806296. https://doi.org/10.1002/adma.201806296.

(2) Tran, A.-T.; Huet, F.; Ngo, K.; Rousseau, P. Artefacts in Electrochemical Impedance Measurement in Electrolytic Solutions Due to the Reference Electrode. *Electrochimica Acta* **2011**, *56* (23), 8034–8039. https://doi.org/10.1016/j.electacta.2010.12.088.

(3) Newman, J. Current Distribution on a Rotating Disk below the Limiting Current. *Journal of The Electrochemical Society* **1966**, *113* (12), 1235. https://doi.org/10.1149/1.2423795.

(4) Antipin, D.; Risch, M. Calculation of the Tafel Slope and Reaction Order of the Oxygen Evolution Reaction between pH 12 and pH 14 for the Adsorbate Mechanism. *Electrochemical Science Advances* **2023**, *3* (6), e2100213. https://doi.org/10.1002/elsa.202100213.

(5) Mefford, J. T.; Zhao, Z.; Bajdich, M.; Chueh, W. C. Interpreting Tafel Behavior of Consecutive Electrochemical Reactions through Combined Thermodynamic and Steady State Microkinetic Approaches. *Energy Environ. Sci.* **2020**, *13* (2), 622–634. https://doi.org/10.1039/C9EE02697E.
